# MOMENTARY EMOTION REGULATION ACROSS HASSLE DOMAINS

**DOI:** 10.1093/geroni/igac059.500

**Published:** 2022-12-20

**Authors:** Jennifer Bellingtier, Gloria Luong, Cornelia Wrzus, Gert Wagner, Michaela Riediger

**Affiliations:** Friedrich Schiller University Jena, Jena, Thuringen, Germany; Colorado State University, Fort Collins, Colorado, United States; Ruprecht Karls University Heidelberg, Heidelberg, Baden-Wurttemberg, Germany; Max Planck Institute for Human Development, Berlin, Berlin, Germany; Friedrich Schiller University Jena, Jena, Thuringen, Germany

## Abstract

Adapting emotion-regulation strategy use to flexibly match contextual features of a hassle is thought to aid in effective coping. We investigated if hassle domains are a pertinent feature for understanding emotion-regulation strategy selection in the everyday lives of adolescents through older adults. Participants, ranging from 14 to 88 years old (N = 325), completed an experience sampling study of approximately 9 days over a 3-week period. At each momentary assessment, participants reported on their affect, hassles, and emotion-regulation strategies. Our findings indicated that strategy use varied by domain. For example, distraction was most common in the health domain, whereas emotion expression was least likely to be used at work or school. More strategies were used when hassles were associated with multiple domains. However, greater domain differentiation was not associated with reduced hassle reactivity. Findings were similar across ages suggesting domains may similarly relate to strategy selection across the lifespan.

